# Does Lipocalin-2 Affect Metabolic Syndrome in Hepatic Infections?

**DOI:** 10.7759/cureus.10040

**Published:** 2020-08-26

**Authors:** Waqas Shahnawaz, Nawal Suhail, Muhammad Ahsan Iqbal Siddiqui, Saira Yasmeen, Syeda Sadia Fatima

**Affiliations:** 1 Biological and Biomedical Sciences, Aga Khan University Medical College, Karachi, PAK; 2 Physiology, Jinnah Postgraduate Medical Centre, Karachi, PAK; 3 Biological and Biomedical Sciences, Aga Khan University, Karachi, PAK

**Keywords:** lipocalin, obesity, hepatitis, diabetes

## Abstract

Background and objective

Lipocalin-2 (LCN-2) is an adipokine that plays a protective role in various inflammatory disorders and regulates innate immune response to acute and chronic infections. However, scant information is available regarding the relationship between serum LCN-2 levels and type 2 diabetes mellitus (T2DM) occurring concurrently with chronic hepatic infections. The present study sought to investigate the association of LCN-2 with T2DM patients with hepatic infections.

Methods

The association of LCN-2 with T2DM, hepatic steatosis, and inflammation was tested in 37 non-T2DM noninfectious individuals (group A, control group) and 55 age-matched patients with T2DM and chronic infection (group B). Anthropometric data were measured and the body-fat percentage was calculated using bioelectrical impedance analysis (BIA). Hemoglobin (Hb), fasting plasma glucose (FPG), hemoglobin A1c (HbA1c), liver function enzymes (LFEs), lipid profile, and total leukocyte count (TLC) were measured. Serum LCN-2 levels were measured using a commercially available sandwich enzyme-linked immunosorbent assay method.

Results

Levels of LCN-2 were significantly elevated in group B (1896.90 ± 73.13 ng/ml) versus control group A (263.58 ± 15.66 ng/mL; p<0.001). LCN-2 correlated moderately with alanine aminotransferase (ALT) (r=0.369), alkaline phosphatase ALP (r=0.419), and HbA1c (r=0.341) (p<0.01). All correlations were lost when adjusted for the presence of hepatitis, indicating that liver infection exacerbates insulin resistance.

Conclusion

Based on our findings, circulating LCN-2 is elevated in T2DM subjects with hepatitis B co-infection and may contribute towards deranged inflammatory response.

## Introduction

Metabolic syndrome (MetS) is a group of disorders that encompasses hypertension, cellular resistance to insulin, central obesity, dyslipidemia, and pro-thrombotic inflammatory states [1]. These disorders are all usually interconnected, and they increase the risk of contracting cardiovascular diseases (CVDs). Although there are several definitions for MetS, those enumerated by the World Health Organization (WHO), the National Cholesterol Education Program’s Third Adult Treatment Panel (NCEP-ATP III), and the International Diabetes Foundation (IDF) are the most widely used and accepted ones [1-2]. The NCEP-ATP III definition has been proposed to be superior to all other existing definitions in diagnosing MetS and, as such, is now the most frequently adopted definition [2]. Worldwide, the prevalence of MetS varies from as low as <10% to as high as >84% based on geographical location, with approximately 25% of the world’s population estimated to be affected by the condition [3]. Therefore, the disease is a major contributor to mortality and general ill-health across the globe. In particular, densely populated Asian countries such as China and India suffer from a high prevalence of MetS. Several studies have been conducted in Pakistan over the years to investigate the prevalence of MetS, out of which a study conducted in 2004 in Karachi, the largest metropolitan area of Pakistan, found a prevalence of 49% in a sample size of 360 [4]. Additionally, four other separate studies have reported a MetS prevalence of 14.95%, 35%, 63.7%, and 22.95%, respectively, making Pakistan one of the countries most affected by MetS worldwide [5-8].

Similar to MetS, type 2 diabetes mellitus (T2DM) is a vast mixture of interconnected metabolic disorders that includes insulin resistance, unique dyslipidemia, obesity, and hyperglycemia [9], with the most common etiological factor being obesity [10], which results in an increased blood glucose concentration [11]. A systematic review conducted in December 2016 to investigate the disease burden and prevalence of T2DM in both rural and urban areas of Pakistan reported a mean prevalence of 11.7% with the prevalence being greater in men than in women and higher in urban than in rural areas [12]. Hence, it is evident that the disease burden of T2DM needs to be controlled in order to reduce the prevalence of a disorder that is leading to severe complications and directly contributing to the prevalence of MetS. The pathophysiology behind MetS is an impaired response of the body to insulin, which normally suppresses the production of glucose and very-low-density lipoprotein (VLDL) [13]. This impairment causes substantial changes in the liver, leading to numerous complications. Hepatic fibrosis occurs due to the overproduction of glucose, cholesterol, and VLDL, which, if left untreated, can eventually lead to steatohepatitis and hepatic cirrhosis [14].

Lipocalins (LCN) are a diverse family of minuscule extracellular secreted proteins that were thought to function as transport proteins in the past. They show great diversity in structure and have now been recognized to participate in a variety of functions including macromolecular complexation, pheromone activity, olfaction, gustation, coloration, prostaglandin synthesis, immune modulation, cell regulation, and as carrier proteins in the clearance of exogenous and endogenous compounds [15]. Our focus is on LCN-2 or neutrophil gelatinase-associated lipocalin (NGAL), which is a glycoprotein derived from adipose tissue. It is also believed to be an inflammatory modulator and contributes to metabolic homeostasis [16]. LCN-2 has been found to be closely linked to obesity, increased blood glucose, and insulin resistance [17]. In addition, it has also been found to be significantly higher during phases of liver injury [18]. This study aimed to investigate the relationship between LCN-2 and hepatic infections in T2DM patients.

## Materials and methods

Study design and patient population

**Figure 1:**
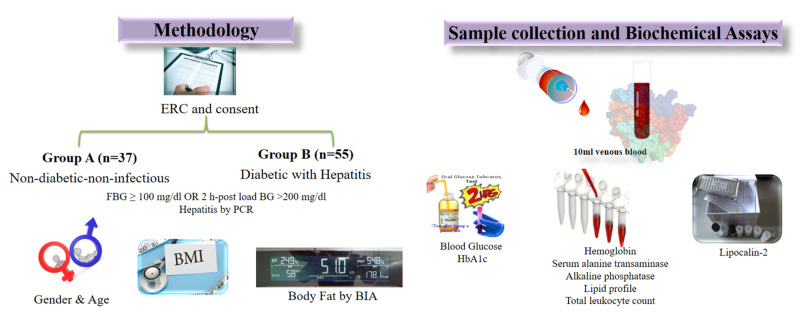
Summary of materials and methods PCR: polymerase chain reaction; BMI: body mass index; BIA: bioelectrical impedance analysis; HbA1c: glycated hemoglobin

This study was conducted at the Aga Khan University (AKU) in collaboration with the diabetic clinic of Jinnah Postgraduate Medical Centre (JPMC). Approval from institutional ethical committees of AKU and JPMC was obtained before initiating the study (ERC-3597-BBS-17 and No.F.2.81-IRB/2017/GENL/419/JPMC, respectively). The study design was cross-sectional, for which a total of 92 subjects were recruited, and these subjects were classified according to their diabetic status. Non-T2DM and noninfectious individuals (n=37) were labeled as group A (control group). while patients with T2DM and chronic infection (n=55) were labeled as group B (disease group). To select the participants, a convenience sampling technique was employed. Participants were only considered to have T2DM if the fasting plasma glucose (FPG) was either more than or equivalent to 100 mg/dl and were considered infectious if the hepatitis B antigen result was positive on patients' medical record cards. Patients with other etiologies of infections (other than hepatic infections) were excluded from the study. Patients who presented to the outpatient department of the diabetic clinic at JPMC and fulfilled the eligibility criteria were enrolled after taking informed consent. Gender, age, weight, and height of the patients were noted and body mass index (BMI) was calculated. Furthermore, body fat was calculated using bioelectrical impedance analysis (BIA). A 10-ml venous blood sample was obtained from each participant after a fasting period of 12-14 hours. Hemoglobin (Hb), hemoglobin A1c (HbA1c), FPG, serum alanine transaminase (ALT), alkaline phosphatase (ALP), lipid profile, and total leukocyte count (TLC) were measured by Hitachi/Cobas automated analyzer (Hitachi, Tokyo, Japan/Roche, Basel, Switzerland). LCN-2 was measured by a sandwich enzyme-linked immunosorbent assay method using LCN-2 (kit cat number 96581 Glory Science, Belgium) (Figure 1).

Sample size and statistical analysis plan

Calculations were done using OpenEpi version 3 software. At 95% confidence interval and 5% absolute precision, the sample size was calculated to be 92 considering the prevalence of T2DM in Pakistan and the anticipated frequency as 50% (unknown anticipated frequency). Data obtained was coded in SPSS Statistics version 21 (IBM, Armonk, NY). Single data entry with visual comparison was performed to minimize error. The distribution of continuous variables was determined. Furthermore, the means and standard deviations were reported respectively. For categorical variables, descriptive analysis was presented in terms of frequencies and percentages. The Mann-Whitney U test for variables that were quantitative, Pearson Chi-Square test for categorical variables, and the Pearson correlation were applied as appropriate. Data were analyzed using SPSS Statistics version 21 and a p-value of less than 0.01 was considered significant.

Operational definition

The NCEP-ATP III criteria [1] defines MetS as the presence of any three of the following five traits: 1. abdominal obesity defined as a waist circumference of ≥102 cm (40 in) in men and ≥88 cm (35 in) in women; 2. serum triglyceride levels of ≥150 mg/dL (1.7 mmol/L) or drug treatment for elevated triglycerides; 3. serum high-density lipoprotein (HDL) cholesterol of <40 mg/dL (1 mmol/L) in men and <50 mg/dL (1.3 mmol/L) in women or drug treatment for low HDL cholesterol; 4. blood pressure (BP) of ≥130/85 mmHg or drug treatment for elevated BP; 5. FPG of ≥100 mg/dL (5.6 mmol/L) or drug treatment for elevated blood glucose.

## Results

No notable differences were observed with respect to age, gender, and BMI between the two groups. Both groups were age-, gender-, and BMI-matched (p>0.05) (Table 1). Body fat and diastolic BP were found to be elevated in group B as compared to group A. Furthermore, liver function enzymes (LFEs) were raised in group B.

**Table 1:** Demographic and biochemical parameters HbA1c: glycated hemoglobin

Variable	Group A (n=37)	Group B (n=55)	P-value
Age (years)	31.86 ± 13.8	34.69 ± 9.46	0.05
Gender	M = 20; F = 17	M = 29; F = 26	-
Body mass index (kg/m^2^)	21.59 ± 3.19	22.67 ± 3.71	0.052
Body fat (%)	19.7 ± 0.963	22.88 ± 1.00	<0.05
Diastolic blood pressure (mmHg)	79.85 ± 2.33	91.22 ± 1.02	<0.05
HbA1c	5.23 ± 0.38	8.52 ± 1.82	<0.05
Serum alanine aminotransferase (IU/L)	14.43 ± 4.09	40.25 ± 5.562	<0.05
Serum alkaline phosphatase (IU/L)	140.46 ± 23.89	262.95 ± 35.31	<0.05
Triglyceride (mg/dl)	134.30 ± 51.19	166.26 ± 28.03	<0.05
Low-density lipoprotein (mg/dl)	79.44 ± 38.49	116.52 ± 9.62	<0.05

**Figure 2:**
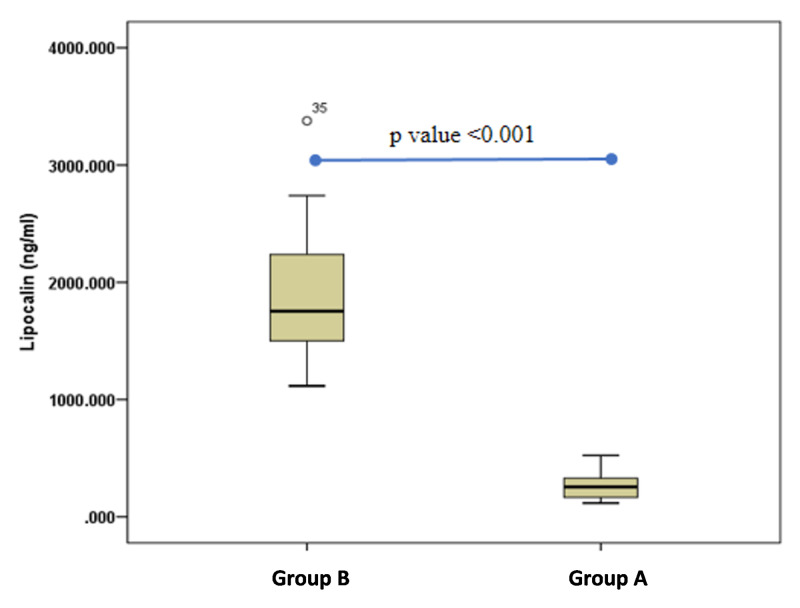
LCN-2 levels for group A and group B Box-plot graph for LCN-2 levels showing results of the Mann-Whitney U test for both groups. Group B had significantly higher levels of LCN-2 with a significant p-value of less than 0.01 LCN-2: lipocalin-2

LCN-2 levels were compared for both the groups, and the results showed significantly raised levels of LCN-2 (p<0.001) in group B (1896.90 ± 73.13 ng/ml SEM) compared to group A (263.58 ± 15.66 ng/ml SEM) (Figure 2). Lastly, LCN-2 levels were evaluated and their correlation with biochemical study parameters was performed using Pearson correlation as shown in Table 2. The results showed that LCN-2 correlated moderately with T2DM markers and LFEs. For T2DM markers, LCN-2 correlated moderately with fasting blood glucose (FBG) (r=0.377) and HbA1c (r=0.341) (p<0.001). Simultaneously, for LFEs, LCN-2 correlated moderately with ALT (r=0.369) and ALP (r=0.419) (p<0.001).

**Table 2:** Pearson correlation of LCN-2 levels with study parameters LCN-2: lipocalin-2; HbA1c: glycated hemoglobin; HbsAg: hepatitis B surface antigen

Variable	r-value	P-value
Fasting blood glucose (mg/dl)	0.377	0.000
HbA1c	0.341	0.001
Alanine animotranferase	0.369	0.000
Alkaline phosphatase	0.419	0.000
HbsAg	-0.0164	0.233
Cholesterol (mg/dl)	0.252	0.034
Triglycerides (mg/dl)	0.279	0.019
Low-density lipoprotein (mg/dl)	0.236	0.050

## Discussion

There are multiple associations with regard to the causes of T2DM, and abdominal obesity has been found to be the leading cause of insulin resistance. Apart from causing T2DM, the associated disrupted balance of hormones and glucose, i.e., the raised levels of insulin and glucose along with elevated adipocyte cytokines, is thought to lead to abnormal lipid profiles, functional atherosclerosis, hypertension, and vasculitis, all of which contribute towards development and advancement of CVDs [19-21]. New theories about the character of adipose tissue and the cytokines released by them have suggested correlations between adipokines such as LCN-2 and their role in preventing systemic insulin resistance and, thus, MetS. However, excess adipose tissue can alter the regulation of homeostasis, inflammation, and insulin resistance and therefore lead to the aforementioned diseases [22,23].

Furthermore, it has been suggested that the constant state of inflammation due to increased adipose tissue and increased level of cytokines initiate multiple processes in the body, which become a leading cause of augmentation and evolution of atherosclerosis [24,25]. Patients suffering from T2DM are prone to developing multiple diseases as complications; however, most of the progression stems from baseline physiological damage caused by the biochemical and hormonal imbalance. There have been multiple explanations as to why there is endothelial damage in diabetics. Apart from being in a constant inflammatory state, patients with T2DM also go through oxidative stress, both in turn leading to disturbance of the vasoprotective nitric oxide (NO) pathway [26]. The basic thrust of this study was to evaluate subjects with both T2DM and hepatic infections with regard to LCN-2, which is a cytokine that was previously shown to be an indicator of the progression of the disease. Moderate correlation of LCN-2 with liver functions such as ALT (r=0.369) and ALP (r=0.419) was seen in the current study, confirming a positive relationship between LCN-2 levels and chronic infection. In a previous study conducted in Germany in 2013 to assess the protective effects of LCN-2 in acute liver damage, it was concluded that increased levels of LCN-2 are indicative of liver damage and correlate with inflammation present in the liver [27].

Furthermore, in addition to finding the links between the adipokine levels and infections, this study also revealed increasing levels of LCN-2 in patients presenting with T2DM and dyslipidemia. Researchers have evaluated the role of LCN levels with regard to HbA1c levels in a few studies previously. A study conducted in Khartoum, Sudan found a positive correlation (p<0.001) between LCN-2 and HbA1c as the latter’s levels were significantly elevated, compared to the controls [28]. Raised LDL levels along with higher total cholesterol and serum creatinine were also observed in disease-positive patients in comparison with controls (p<0.02).

We found an interrelation between LCN-2 levels and HbA1c (r=0.341) (p<0.001). LDL levels were also found to be raised in the patient group; however, a significant association could not be established in this regard. In multiple studies conducted previously, it has been established that LCN-2 levels do correlate or contribute towards the development of heart disease or cardiac hypertrophy. In a total cohort study conducted by Wu et al. in 2014, it was demonstrated that LCN-2 levels had a positive relationship with age, BMI, and waist circumference. Other variables measured to evaluate T2DM such as FBG, post-two-hour glucose load, insulin resistance (homeostasis model), and HbA1c also showed associations. Lipid profile and BP along with glomerular filtrate rate (eGFR) were also positively linked [29]. Of all these factors, BMI, waist circumference, FBG, and diastolic BP were considered to evaluate MetS-like characteristics in our study.

It is evident from this study that patients who suffer from T2DM and chronic infections have significantly elevated levels of serum LCN-2. However, our study is limited by the small sample size that we were able to recruit. Yet, the trend shown in this pilot study showcases LCN-2 levels as a diagnostic tool to identify the risk in individuals who are not yet suffering from MetS but are on the verge of getting it. The risk factors could be family history, central obesity, or a sedentary lifestyle, where LCN-2 levels will provide an idea of the progression of the risk. Similarly, in individuals diagnosed with MetS, LCN-2 levels will provide an idea of the progression of the disease as well.

## Conclusions

It may be concluded that high levels of circulating LCN-2 in subjects presenting with both T2DM and hepatitis B may lead to a deranged inflammatory response. LCN-2 has proved to be a reliable marker for multiple disease manifestations, such as T2DM, atherosclerosis, dyslipidemia, and liver damage. There are protective qualities attributed to LCN-2 as well, which, once utilized in future research, can be used to manipulate LCN-2 as a key factor in immune modulation. The positive correlations can be used to further research and evaluate where to consider LCN-2 as a considerable factor in risk factor assessment and management of T2DM superimposed with hepatitis. Assessing the confounding factors such as race could be considered in order to establish new universal guidelines regarding the use of cytokines, including LCN-2.
